# Mutations underlying Episodic Ataxia type-1 antagonize Kv1.1 RNA editing

**DOI:** 10.1038/srep41095

**Published:** 2017-02-20

**Authors:** Elizabeth A. Ferrick-Kiddie, Joshua J. C. Rosenthal, Gregory D. Ayers, Ronald B. Emeson

**Affiliations:** 1 Department of Molecular Physiology & Biophysics, Vanderbilt University School of Medicine, Nashville, TN 37232, United States; 2 Eugene Bell Center for Regenerative Biology and Tissue Engineering, The Marine Biological Laboratory, Woods Hole, MA 02543, United States; 3 Institute of Neurobiology, University of Puerto Rico Medical Sciences Campus, San Juan, PR 00901, United States; 4 Center for Quantitative Sciences, Vanderbilt University Medical Center, Nashville, TN 37232, United States; 5 Department of Pharmacology, Vanderbilt University School of Medicine, Nashville, TN 37232, United States

## Abstract

Adenosine-to-inosine RNA editing in transcripts encoding the voltage-gated potassium channel Kv1.1 converts an isoleucine to valine codon for amino acid 400, speeding channel recovery from inactivation. Numerous Kv1.1 mutations have been associated with the human disorder Episodic Ataxia Type-1 (EA1), characterized by stress-induced ataxia, myokymia, and increased prevalence of seizures. Three EA1 mutations, V404I, I407M, and V408A, are located within the RNA duplex structure required for RNA editing. Each mutation decreased RNA editing both *in vitro* and using an *in vivo* mouse model bearing the V408A allele. Editing of transcripts encoding mutant channels affects numerous biophysical properties including channel opening, closing, and inactivation. Thus EA1 symptoms could be influenced not only by the direct effects of the mutations on channel properties, but also by their influence on RNA editing. These studies provide the first evidence that mutations associated with human genetic disorders can affect *cis*-regulatory elements to alter RNA editing.

The Kv1.1 voltage-gated potassium (Kv) channel α-subunit plays an important role in regulating neuronal excitability. By dampening excitability at the axon initial segment and juxtaparanodal region^1^, it can influence action potential initiation, propagation and reduce nerve terminal excitability, permitting fine-tuning of neurotransmitter release^2^. Underscoring its physiological importance, genetic knockout studies have revealed that mice lacking Kv1.1 expression develop spontaneous seizures, hyperalgesia, and neurogenic cardiac dysfunction^3^,^4^,^5^. Although Kv1.1 can form functional homotetrameric channels, Kv1.1 is predominantly found in heterotetramers with other Kv1 family members that contribute to a large diversity of Kv1 channel kinetics and pharmacology throughout the mammalian central nervous system^6^,^7^,^8^,^9^. Further regulation of Kv1 channels is mediated through co-assembly with four accessory Kvβ-subunits^10^,^11^, where an inactivating domain in the Kvβ amino terminus binds to the inner vestibule of the channel pore to block current flow, a process known as fast (N-type) inactivation^12^,^13^.

RNA transcripts encoding Kv1.1 are modified by a site-specific adenosine-to-inosine (A-to-I) RNA editing event in which a genomically-encoded isoleucine (AUU) is converted to a valine (IUU) codon at amino acid position 400 of the protein^14^. This amino acid lies within the S6 transmembrane domain predicted to line the ion-conducting pore of the channel. Editing of Kv1.1 transcripts is dependent upon a region of double-stranded RNA (dsRNA) formed by intramolecular base-pairing interactions between imperfect, inverted repeat elements surrounding the targeted adenosine moiety^15^. This process is catalyzed by ADAR2, a member of a family of dsRNA-specific adenosine deaminases (ADARs)^15^,^16^. Previous studies have revealed that Kv1.1 channels containing edited [Kv1.1(V)] subunits display a 20-fold faster rate of recovery from Kvβ1.1-inactivation, compared to non-edited channels [Kv1.1(I)]^15^. More recent studies have indicated that only small alterations in the hydrophobicity of the editing site amino acid were needed to reproduce the editing-dependent alterations in recovery kinetics^17^.

Episodic ataxia type-1 (EA1) is a sporadic neurological disorder characterized by stress-induced motor discoordination, involuntary, repetitive muscle contraction (myokymia), and can coincide with seizures^18^. Genetic linkage analyses have identified over 30 heterozygous mutations within the gene encoding human Kv1.1 (*KCNA1*) that have been associated with this disorder^19^. The majority of EA1-related mutations result in a loss of channel function, reduced surface expression, or a change in biophysical properties where the mutant subunits can exert a dominant-negative effect by association with wild-type α-subunits^19^,^20^,^21^,^22^,^23^,^24^,^25^,^26^. Despite the broad distribution and altered function resulting from these mutations throughout the channel protein, no clear correlation has been established between the diverse clinical phenotypes of EA1 patients and specific mutations within Kv1.1^18^,^19^,^20^,^21^,^22^,^23^,^24^,^25^,^26^,^27^. This lack of consistency in clinical presentation suggests that additional mechanisms could also be responsible for alterations in channel function and EA1 heterogeneity.

At least three EA1-associated mutations (V404I, I407M, and V408A) have been identified within the duplex region required for editing of Kv1.1 RNAs^20^,^21^,^22^,^23^,^28^,^29^. Due to the proximity of the mutant amino acids to the editing site (I400 V), we sought to determine whether such EA1 mutations affect the editing profiles for Kv1.1 transcripts and to determine the resultant biophysical properties of edited, mutant channels. Our studies reveal an antagonistic relationship between these EA1 mutations and RNA editing. Each EA1 mutation decreased the rate of RNA editing using an *in vitro* editing system and the V408A mutation decreased the extent of editing in a previously characterized mouse model of EA1. Furthermore, we show that editing can have a variable effect on each type of EA1 mutant protein, altering voltage sensitivity, activation and deactivation, and inactivation with a Kvβ subunit. Not only do our studies demonstrate that EA1 mutations can impede RNA editing and alter resulting protein function, but also represent the first examples of existing disease-associated human mutations which act to disrupt *cis*-regulatory elements required for RNA editing.

## Results

### EA1-associated mutations alter RNA editing *in vitro*

**Figure 1:**
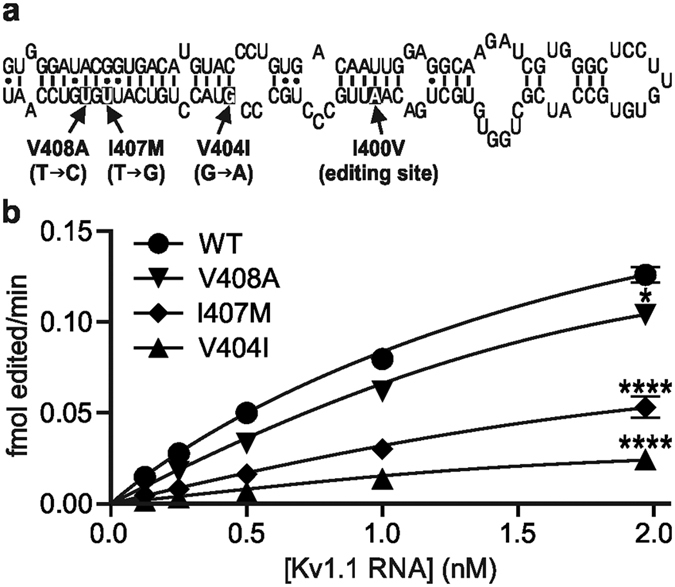
Quantitative analysis of *in vitro* RNA editing rates for wild-type and mutant Kv1.1 transcripts. (**a**) The predicted secondary structure for a portion of the wild-type (WT) Kv1.1 pre-mRNA is indicated with the positions of the A-to-I editing site (I400 V) and three non-synonymous mutations associated with EA1 shown with inverse lettering. (**b**) Wild-type and mutant Kv1.1 RNA minigenes, encompassing the duplex region required for editing, were *in vitro* transcribed and incubated with nuclear extracts prepared from HEK293 cells transiently expressing rat ADAR2. The extent of editing was quantified by high-throughput sequence analysis as described previously^33^ and used to calculate the editing rate. Single exponential curves were fitted to the data for emphasis. Statistical differences were determined for replicates at the 2 nM RNA concentration (mean ± SEM, n = 4 replicate reactions, *p ≤ 0.05; ****p ≤ 0.0001). Small error bars were obscured by the data symbols in some cases.

Three known EA1-associated mutations, V404I, I407M, and V408A, lie within the predicted 114-bp RNA duplex, which represents the minimum sequence required for site-specific editing of Kv1.1 transcripts by ADAR2 (Fig. 1a)^15^. Using an RNA-folding algorithm (mfold)^30^, we examined whether any of these mutations were predicted to grossly alter the structure of the duplex region. Results from this analysis revealed that each individual mutation predicted a single-nucleotide mismatch within the duplex at each mutation site, with no further perturbations to the predicted RNA secondary structure and only minimal alterations in the free-energy (ΔG) calculations for each duplex (*data not shown*)^31^. To test whether these mutations affected the rate of editing for Kv1.1 RNAs, each of the EA1-associated mutations was incorporated separately into constructs encompassing a 463-bp region centered on the known editing site. RNA transcripts were transcribed *in vitro* using these minigenes as a template and a range of concentrations for each RNA was subjected to an *in vitro* editing assay using ADAR2 protein derived from nuclear extracts isolated from HEK293 cells transiently expressing ADAR2^32^. The extent of editing was quantified by high-throughput sequence analysis as described by Hood *et al*.^33^ and used to calculate the rate of editing (Fig. 1b). Results from these studies clearly demonstrated that introduction of any of these EA1-associated point mutations into the wild-type sequence was sufficient to decrease the editing rate for Kv1.1 transcripts *in vitro*. Furthermore, the magnitude of this rate decrease corresponded to the proximity of the mutation to the editing site (I400 V), with the most severe deficit observed for the V404I mutation (81% rate reduction at 2 nM RNA), and a 58% and 17% reduction in editing rate for the I407M and V408A mutations, respectively.

### A mouse model of EA1 (V408A/+) alters RNA editing *in vivo*

**Figure 2:**
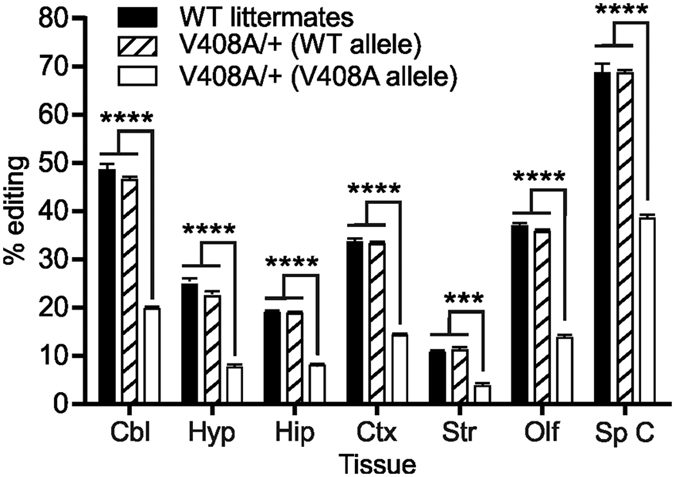
Quantitative analysis of allele-specific Kv1.1 editing in V408A mutant mice. The extent of editing for the wild-type and mutant alleles in heterozygous V408A adult mice (V408A/+), compared to wild-type littermates, was determined for RNA isolated from dissected brain regions and spinal cord by high-throughput sequence analysis (mean ± SEM, n = 4, ***p ≤ 0.001, ****p ≤ 0.0001). Cbl, cerebellum; Hyp, hypothalamus; Hip, hippocampus; Ctx, cortex; Str, striatum; Olf, olfactory bulb; Sp C, spinal cord.

To date, only one mouse model of EA1 has been developed^34^. Mutant mice homozygous for the V408A allele die between embryonic day 3 (E3) and E9, whereas V408A/+ heterozygotes are characterized by stress-induced ataxia as well as attenuated cerebellar Purkinje signaling, which has been attributed to action potential broadening at basket cell boutons leading to increased GABA release^34^,^35^. To determine whether the presence of the V408A mutation inhibited Kv1.1 editing *in vivo*, we isolated RNA from multiple dissected brain regions and spinal cord of wild-type and V408A/+ mutant animals to determine RNA editing profiles by high-throughput sequence analysis of Kv1.1 transcripts. Since this deep-sequencing approach generates sequence reads covering both the V408A mutation and the editing site, it was possible to quantify allele-specific editing profiles in V408A/+ heterozygotes. Results from this analysis indicated that the extent of editing for the wild-type allele in V408A/+ mutant mice was similar to that observed in wild-type animals. Editing for the mutant V408A allele showed a 59% reduction in site-specific editing efficiency in all tissues examined when compared to either wild-type littermates or the V408A/+ wild-type allele (Fig. 2).

### Gating properties are altered between non-edited and edited Kv1.1 channels harboring EA1 mutations

All three EA1 mutations can affect the rate of editing *in vitro* and the V408A allele can reduce the extent of editing *in vivo* (Figs 1b and 2). Although editing and EA1 mutations separately have been shown to alter the biophysical properties of Kv1.1 channels, it is unknown whether editing may cause unique effects when paired with these EA1-associated mutations. Similarly, it is unclear whether the phenotypic alterations observed in patients bearing the V404I, I407M, or V408A mutations result from changes in channel function mediated by these missense mutations alone or in concert with their affects upon editing. To address these questions, *Xenopus* oocytes were injected with *in vitro* transcribed RNAs encoding either the non-edited (N) or edited (E) isoforms of the wild-type, V404I, I407M, or V408A Kv1.1 subunits, expressed as homotetramers.

**Figure 3:**
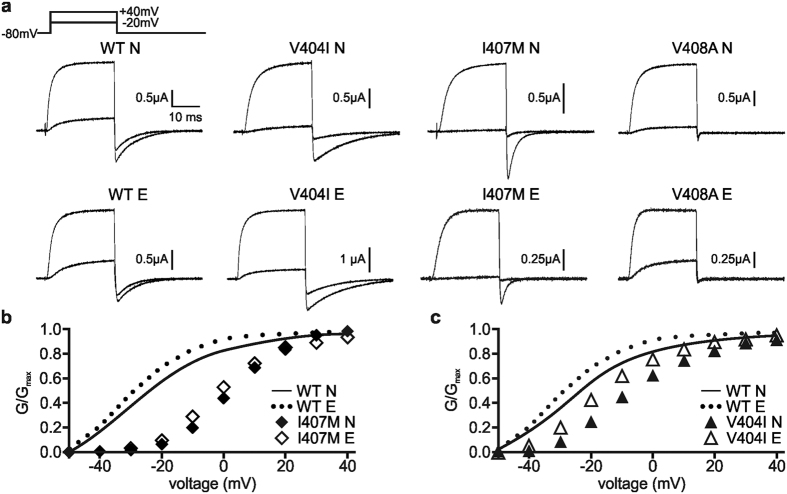
Voltage-dependence of non-edited compared to edited mutant channels. Whole-cell K^+^ currents were recorded from oocytes expressing either the non-edited (N) or edited (E) isoforms of homotetrameric wild-type (WT), V404I, I407M, or V408A Kv1.1 channels. Test potentials were elicited in 10 mV voltage steps from −50 to 40 mV, from a holding potential of −80 mV. (**a**) Representative activating traces at −20 and 40 mV are shown for each construct. (**b**,**c**) Conductance (G) versus voltage plots are shown where data have been normalized to the maximal conductance (Gmax), demonstrating shifts in voltage dependence for (**b**) I407M and (**c**) V404I (mean ± SEM, n = 4–8 oocytes). Normalized conductance was measured from tail current amplitude. Small error bars were obscured by the data symbols.

The voltage dependence of activation for each channel subtype was analyzed to determine editing-dependent changes, and representative traces for each channel are shown in Fig. 3a. The relationship between macroscopic conductance and voltage was quantified for each channel type. For most constructs, this was derived from normalized tail current measurements; however, V408A E closed too quickly for accurate measurements of tail currents so conductance was measured using outward currents (*see* equation (1) *in Materials & Methods*). Conductance (G) versus voltage (V) curves were fit to a Boltzmann function, equation (2),


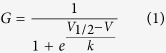


to estimate the midpoint of channel activation (V_1/2_) and the relative voltage sensitivity (k) (Table 1). Consistent with previous reports for I407M N channels^22^, we observed a 30 mV positive shift in the V_1/2_, but this change was not influenced by editing (Fig. 3b and Table 1). By contrast, V404I also caused a positive shift, but it was more pronounced for the non-edited channel (a shift of 22.1 mV for the non-edited channel, 14.2 mV for the edited channel; Fig. 3c and Table 1). Thus, editing partially ameliorated the alteration in channel function caused by the V404I mutation. V408A channels did not exhibit altered voltage-dependence for either the non-edited or edited isoforms (Supplementary Fig. S1 and Table 1). Editing had little effect on voltage sensitivity for any of the wild-type or mutant homotetrameric channels (Table 1).

**Figure 4:**
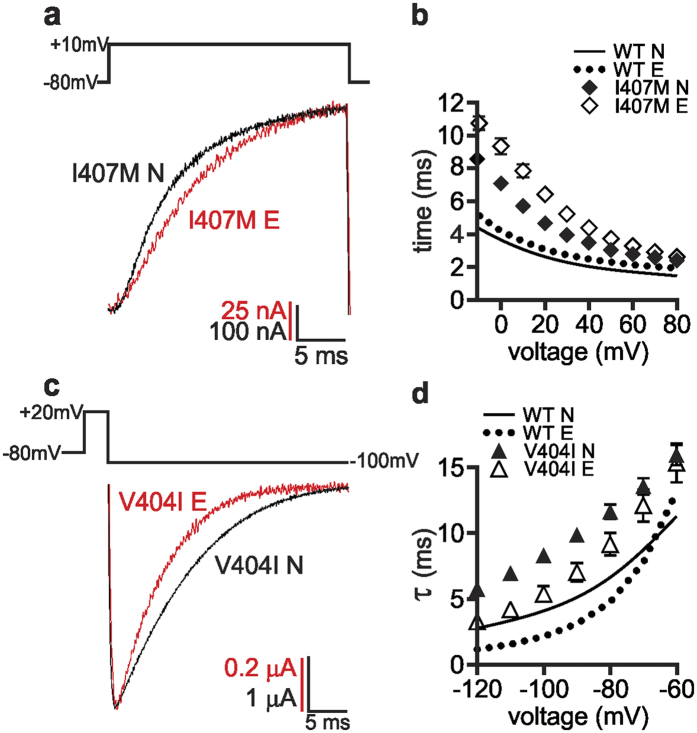
Editing alters gating kinetics of I407M and V404I channels. (**a**) Representative activation traces, depicting whole-cell currents, were recorded from oocytes expressing either the I407M N or I407M E channel. Test potentials were elicited in 10 mV voltage steps from −10 to 80 mV, from a holding potential of −80 mV. (**b**) Activation kinetics were measured as the time to reach half-maximal current amplitude (mean ± SEM, n = 3–7 oocytes). I407M N and I407M E channels were significantly slowed in their time to half-activation compared to each other, in the voltage range −10 to 70 mV (0.05 > p ≥ 0.0008). I407M N was significantly slower than WT N at all voltages (p ≤ 0.0001) and I407M E was significantly slower than WT E at all voltages (0.01 > p ≥ 0.0001). (**c**) Representative tail current traces, depicting whole-cell K+ currents, were recorded from oocytes expressing either the V404I N or V404I E channel. Following a holding potential of −80 mV and a depolarizing pulse to 20 mV, test potentials were elicited in 10 mV voltage steps from −120 to −60 mV. (**d**) Closing kinetics were determined by fitting the tail currents with single exponential curves to determine the associated τ value; (mean ± SEM, n = 3–6 oocytes). V404I N channels closed slower than V404I E from −120 to −100 mV (0.05 > p ≥ 0.0066). V404I N channels closed slower than WT N at all voltages (p < 0.0001) and V404I E channels closed significantly slower than WT E channels from −120 to −80 mV (0.01 ≥ p ≥ 0.0005). Small error bars were obscured by the data symbols in some cases.

To examine how editing affected channel opening kinetics, the time to reach half-maximal activation across a range of voltages was determined. The only editing-dependent change was observed for the I407M mutation. Both I407M channels opened more slowly than their wild-type counterparts, but the slowing was more severe for I407M E channels (Fig. 4a,b and Supplementary Fig. S2), leading to channels with an exacerbated slow opening phenotype. In addition, I407M E and V408A E channels demonstrated non-linearity in their voltage dependence for outward currents, particularly at very positive voltages, reaching peak current amplitudes at 50 and 40 mV respectively, with further voltage steps resulting in decreasing current amplitudes (Supplementary Fig. S3).

Closing (deactivation) kinetics were measured by fitting single exponential curves to tail current traces to obtain estimates of tau (τ), the reciprocal of the closing rate constant. Editing resulted in wild-type channels closing slightly faster (Supplementary Fig. S4). In addition, the I407M and V408A mutations greatly increased closing speeds on their own. The editing of I407M channels had only a small effect on deactivation kinetics, while the edited V408A channels closed so quickly that the closing rate could not be accurately measured (Supplementary Fig. S4). Unlike the other mutations, however, V404I led to slower closing speeds and editing partially ameliorated this phenotype (Fig. 4c,d).

Slow inactivation (C-type) was examined by analyzing channel function under conditions of long depolarizations. The I407M E and V408A E channels demonstrated editing-dependent dysfunction, with a prominent fast component of their inactivation appearing alongside the slow component. Thus, while a single exponential function was sufficient to describe the inactivation for the majority of the channels, I407M E and V408A E required a double exponential fit (Supplementary Fig. S5a). Both the fast and slow components of the I407M E and V408A E channels were fast compared to their non-edited counterparts (Supplementary Fig. S5b). By contrast, the extent of inactivation was predominantly mutation-driven, except for the V408A mutation, in which editing decreased the extent of inactivation, bringing it closer to wild-type levels (Supplementary Fig. S5c).

### Inactivation kinetics are altered between non-edited and edited EA1 mutant proteins

Previous studies by Bhalla *et al*.^15^ found that the most profound change in channel function between non-edited and edited isoforms of the wild-type Kv1.1 channel was a change in the rate of recovery from channel inactivation, presumably by altering interactions with an inactivating Kvβ subunit. To determine the effect of EA1 mutations on this biophysical property, non-edited and edited isoforms of the wild-type, V404I, I407M, and V408A channels were co-expressed with Kvβ1.1 to measure N-type, fast inactivation kinetics and recovery from inactivation.

**Figure 5:**
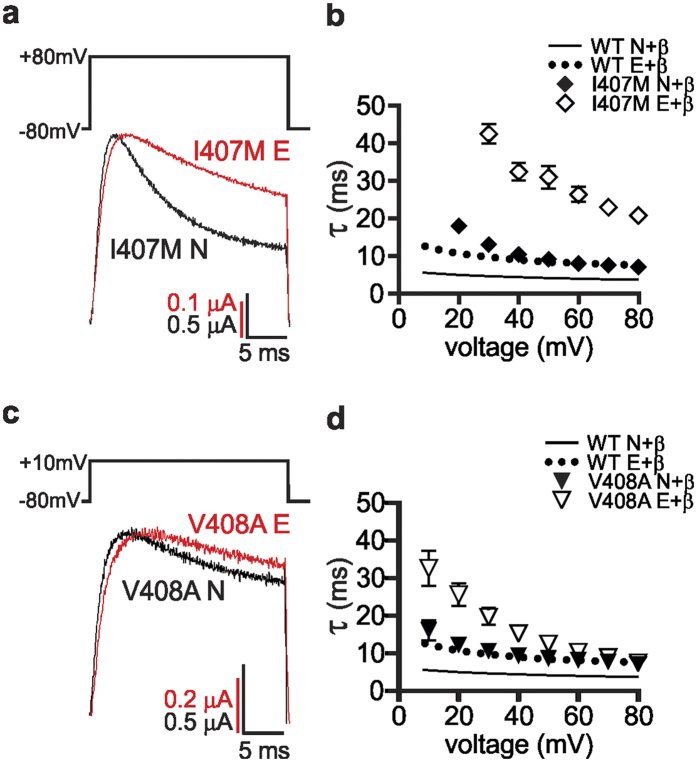
Editing slows Kvβ1.1-induced inactivation kinetics of I407M and V408A channels. (**a**,**c**) Representative β-inactivation traces, depicting whole-cell K^+^ currents, were recorded from oocytes co-expressing the Kvβ1.1 subunit and either the (**a**) I407M or (**c**) V408A channel, in the non-edited (N) or edited (E) isoform. Test potentials were elicited in 10 mV voltage steps from 10 to 80 mV, from a holding potential of −80 mV. (**b**,**d**) Inactivation kinetics were measured by fitting single exponential curves to the test pulse currents, to determine the associated τ value (mean ± SEM, n = 3–6 oocytes). (**b**) I407M E channels were significantly slower to inactivate than I407M N channels at every voltage (p ≤ 0.0001) and both I407M N and I407M E channels were slower than WT N and WT E channels, respectively, at every voltage (p ≤ 0.0001). (**d**) V408A E channels were significantly slower than V408A N channels from 10 to 50 mV (0.05 > p ≥ 0.0005). V408A E channels were slower than WT E channels from 10 to 60 mV (0.05 > p ≥ 0.0001). V408A N channels were significantly slower than WT N channels at all voltages (p ≤ 0.0001). Small error bars were obscured by the data symbols in some cases.

Oocytes expressing each channel subtype, along with Kvβ1.1, were subjected to short depolarizing pulses to different voltages and the resulting fast inactivation traces were fit to a single exponential. These studies identified a previously uncharacterized difference in wild-type channels where editing modestly slowed the rate of channel inactivation (Supplementary Fig. S6). V404I N and E channels inactivated within the wild-type range, without exhibiting any editing-dependent changes (Supplementary Fig. S6). Inactivation for non-edited isoforms of the I407M and V408A mutants resembled edited, wild-type channels, however, editing of I407M and V408A resulted in drastically slower rates of inactivation for both channels (Fig. 5). This effect was most extreme and apparent at all voltages for I407M E, whereas slowing was only observed for V408A E with shallow depolarizations. Interestingly, the V404I N channels also exhibited a low extent of inactivation where inactivation could not be measured in over half the oocytes tested (*data not shown*). This variability in the extent of inactivation for the mutant channels is consistent with previous studies demonstrating that the extent of inactivation could be manipulated by varying the aliphatic amino acid residues at the position of the editing site^17^.

Long depolarizing pulses were measured to determine the fast and slow components of Kv1.1 channels when co-expressed with Kvβ1.1 (Supplementary Fig. S7a). Double exponential curves were fit to the inactivating traces, to determine the fast and slow τ values and the relative amplitude of the fast component of the inactivation (compared to the slow component). The fast and slow τ values largely corresponded to the results described for the β-inactivation of the short pulses and the slow inactivation of the long pulses without Kvβ1.1 (*data not shown*). In wild-type channels, editing led to an increase in the relative amplitude of the fast component of inactivation. An editing-dependent change also was observed for the V404I channels, where editing brought the relative amplitude of the fast component closer to that of the wild-type channel (Supplementary Fig. S7b).

**Figure 6:**
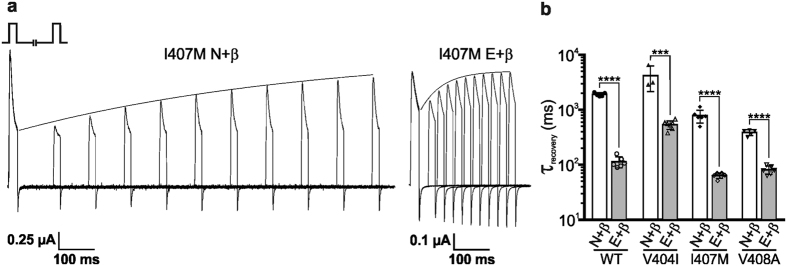
Editing alters the recovery from Kvβ1.1-induced inactivation in V404I, I407M, and V408A channels. Whole-cell K^+^ currents were recorded from oocytes co-expressing the Kvβ1.1 subunit and either a non-edited (N) or edited (E) isoform of the wild-type (WT) or mutant Kv1.1 channel. (**a**) Representative I407M N and I407M E recovery traces are overlaid to depict the increased rate of recovery from β-inactivation, typical of an E isoform. A two-pulse protocol was used, eliciting a depolarizing pulse to 80 mV followed by a variable interpulse duration at −80 mV before a final depolarizing pulse at 80 mV. Recovery from β-inactivation was plotted as the time for the second pulse to regain the current amplitude of the first pulse. (**b**) τ values were determined by fitting single exponential curves to the recovery plots (mean ± SEM, n = 3–7 oocytes, ***p ≤ 0.001, ****p ≤ 0.0001).

Finally, the rate of recovery from fast inactivation was measured using a two-pulse protocol, where the fractional recovery at specific time intervals was assessed after the onset of inactivation. A representative experiment for oocytes expressing either I407M N or E channels is presented in Fig. 6a. As previously reported in Bhalla *et al*.^15^, editing increased the rate of recovery when comparing non-edited and edited isoforms of the wild-type channel (Fig. 6b). All edited isoforms of the mutant channels exhibited a significantly faster rate of recovery than their respective non-edited counterparts (Fig. 6b and Supplementary Table S1). Recovery from inactivation for the V404I E channel was significantly slower than that of the WT E channel, whereas for the I407M N, I407M E, and V408A N channels it was faster compared to its corresponding wild-type channel. Although the extent of the effect differed for each mutation, editing resulted in a unique and substantial contribution to the rate of recovery from fast-inactivation for each channel type.

## Discussion

The conversion of A-to-I by RNA editing has been shown to represent an important post-transcriptional modification by which to modulate the function of numerous proteins critical for nervous system function^36^. Previous studies have shown that site-selective editing of transcripts encoding the Kv1.1 channel can affect the rate of recovery from channel inactivation, the binding of drugs and highly unsaturated fatty acids, the regulation of homotetrameric Kv1.1 channel trafficking, and seizure-susceptibility in chronic epileptic rats^15^,^17^,^37^,^38^,^39^. While numerous EA1-associated mutations have been identified throughout the *KCNA1* coding region, several of these mutations (V404I, I407M, and V408A) are within close proximity to the Kv1.1 editing site (I400 V) and also are predicted to disrupt the critical RNA duplex structure required for this post-transcriptional modification.

To our knowledge, the present studies represent the first demonstration that disease-associated mutations can disrupt critical *cis*-regulatory elements to change their gene’s RNA editing profile, by altering the RNA structure required for site-selective A-to-I conversion. Results using both *in vitro* and *in vivo* model systems have shown significant reductions in the extent and rate of editing for Kv1.1 transcripts harboring specific EA1 mutations (Figs 1b and 2). Importantly, because the wild-type allele RNA was unchanged in the V408A/+ mouse model, it is likely that the observed changes in the editing of the V408A allele-derived RNA were solely due to the V408A mutation and not due to any developmental, compensatory changes. Our studies suggest that both synonymous and non-synonymous duplex-disrupting mutations and single nucleotide polymorphisms within Kv1.1 and other edited RNA targets may also affect the expression of their specific edited isoforms, thus altering the activity of the encoded protein products.

These studies also have revealed that the effects of EA1 mutations on Kv1.1 function are far more complex than originally anticipated, as each mutation produces channels with unique biophysical properties that depend the I400 V amino acid identity, mediated by RNA editing. The V404I mutation altered several electrophysiological parameters on its own, but the edited isoform demonstrated less drastic changes than the non-edited isoform, as observed for channel voltage sensitivity, closing kinetics, and the amplitude of β-inactivation (Figs 3c and 4c,d, Supplementary Fig. S7). Although it is tempting to speculate that editing could dampen the defects in channel function resulting from this point mutation, it also should be noted that this mutation largely prevents the RNA from being edited in the first place (Fig. 1b). Thus, it is anticipated that edited V404I isoforms contribute little to the electrophysiological properties of Kv1.1 channels in those tissue where they are expressed. Unlike the V404I channel, however, editing combined with the I407M or V408A mutations led to more severe channel dysfunctions than the non-edited isoforms. Edited isoforms of both I407M and V408A exhibited unusually slow β-dependent inactivation kinetics (Fig. 5) and severe defects in activation at higher voltages (Supplementary Fig. S3) that could possibly be caused by a significantly faster entry into, or slower recovery from, C-type inactivation (Supplementary Fig. S5)^40^. In addition, while the I407M mutation slowed the kinetics of channel opening, the effect was greater for the edited isoform (Fig. 4a,b). These studies also extended the characterization of the I407M mutation, as previous studies of the non-edited I407M channel reported only alterations in expression and voltage-sensitivity^41^, while the present study also shows changes in kinetics (Fig. 4a,b and 5). Further characterizations of edited EA1 mutant channels could help us better understand their physiological defects *in vivo*. These include stimulating the channels with action potential-like commands (trains of depolarizing pulses) to assess cumulative inactivation, as well as probing the voltage-dependence of their inactivation. Since these mutations also led to decreases in the editing of Kv1.1 transcripts, additional experiments will be required to test the relative contribution of editing-dependent and independent effects, especially when the Kv1.1 proteins are co-assembled into heterotetramers with other Kv1.x family members^20^,^41^,^42^.

Although our studies suggest that edited isoforms of mutant channels represent a smaller portion of the total Kv1.1 population, they may still exert functional effects, particularly in tissues with higher editing levels (such as cerebellum and spinal cord) (Fig. 2). This is supported by previous studies, which have shown that incorporating even one edited subunit into a Kv1.x heterotetramer was sufficient to alter its sensitivity to open-channel blocking molecules^37^. Alternatively, despite the many functional differences observed between edited isoforms of the mutant channels, all recovered from fast inactivation significantly faster than their non-edited counterparts (Fig. 6). As these EA1 mutations reduced their own isoform editing, it is predicted that the overall recovery from fast inactivation *in vivo* will be comparatively slow, possibly resulting in unanticipated effects that could prevent normal neuronal signaling.

While no clear correlation has been established between the diverse clinical phenotypes of EA1 patients and specific mutations within Kv1.1^18^,^19^,^20^,^21^,^22^,^23^,^24^,^25^,^26^,^27^, part of the observed variability in symptoms might be explained by differences in RNA editing. These phenotypic differences could arise from EA1 mutations that disrupt the editing duplex, or from overall changes in Kv1.1 editing regulation. Although the mechanisms regulating Kv1.1 RNA editing are largely unknown, recent studies have demonstrated that inducing rats with chronic epilepsy led to a 4-fold increase in Kv1.1 editing in the entorhinal cortex^38^. Interestingly, once Kv1.1 editing was increased, recordings in isolated rat brain slices demonstrated that these animals had a decreased sensitivity to 4-aminopyridine-induced seizure-like events, suggesting that increasing editing might dampen seizure susceptibility. Similarly, analyses of patients undergoing surgery for mesial temporal lobe epilepsy revealed that having increased levels of Kv1.1 RNA editing was negatively correlated with the period of years that the patients had experienced epileptic activity^43^, suggesting that decreased Kv1.1 editing may represent a risk factor for long-term seizures. Graves *et al*.^27^ clinically surveyed two families containing the same EA1 mutation (F414S), and found that one family exhibited seizures while the other did not, raising the possibility that additional factors, such as differences in editing regulation, could represent an explanation for these phenotypic differences. As previous studies have shown that open-channel blocking drugs interact less with edited Kv1.1 homo- and heterotetramers^37^, a precise therapeutic strategy for the treatment of Kv1.1-dependent seizures may require not only a knowledge of the specific mutation(s) involved, but also the editing profiles of Kv1.1 transcripts.

## Materials and Methods

### Kv1.1 and Kvβ1.1 constructs

A 463 bp-region encompassing the duplex required for Kv1.1 editing was amplified using the polymerase chain reaction (PCR) from human genomic DNA using sense (5′-GCGAAGCTTCCTCTTCATCGGGGTCATCCT-3′) and antisense (5′-GCGGCGGCCGCAGTTTTGGTTAGCAGTGG-3′) oligonucleotide primers in exon 2. To aid in subcloning, the primers incorporated Hind III and Not I restriction sites on their 5′-ends for the sense and antisense primers, respectively. The PCR amplicon was subcloned into the mammalian expression vector, pRc-CMV (Thermo Fisher) to generate a wild-type Kv1.1 minigene. To generate the V404I, I407M, and V408A minigenes, the wild-type Kv1.1 construct was mutagenized using the QuikChange II Site-Directed Mutagenesis kit (Agilent Technologies), where the PCR reactions were supplemented with 5% DMSO. Full-length mouse Kv1.1 (Addgene) and mouse Kvβ1.1 (Thermo Scientific) cDNAs were subcloned into the *Xenopus* expression vector, pGEM HE^44^. The following full-length constructs were created by PCR mutagenesis from the full-length mouse non-edited Kv1.1 cDNA and validated by sequence analysis: wild-type edited Kv1.1 and V404I, I407M, and V408A mutant Kv1.1 (non-edited and edited) cDNAs.

### *In vitro* analysis of RNA editing

RNAs were transcribed *in vitro* from the wild-type Kv1.1 minigene, as well as corresponding minigenes harboring the V404I, I407M, and V408A mutations using the MAXIscript kit (Ambion) with T7 RNA polymerase according to manufacturer’s instructions. Nuclear extracts were prepared from transiently transfected HEK293 cells expressing rat ADAR2, as described previously, and stored at −80 °C until required^32^,^45^. Immediately prior to *in vitro* editing analysis, nuclear extracts were diluted 1:10 in dialysis buffer [20 mM HEPES, 1 mM EDTA, 1 mM EGTA, 10% glycerol, 300 mM NaCl, 1 mM PMSF, 1 mM DTT, 1X complete, EDTA-free protease inhibitor cocktail (Roche)], before a 2-hour incubation at 30 °C with RNase inhibitors and RNA substrates varying in concentration from 0.125 to 2 nM. Nuclear extracts represented one-third of the total 50 μL reaction volume which was diluted with the RNA substrate and water to reduce the glycerol concentration into a range necessary for ADAR2 activity. The incubation time was determined empirically by time-course analyses to ensure that editing of the wild-type Kv1.1 minigene was within the linear range of the reaction (*data not shown*). Reactions were terminated by the addition of TRIzol (Ambion) and RNA was extracted according to the manufacturer’s protocol. RNA was reverse-transcribed with random primers using the High Capacity cDNA Reverse Transcription kit (Applied Biosystems) and the extent of RNA editing was quantified by high-throughput multiplexed sequence analysis as described previously^33^. The editing rate was calculated as the fmol RNA converted to the edited isoform divided by the duration of the reaction.

### *In vivo* analysis of RNA editing

All animal care and experimental procedures involving mice were approved by the Vanderbilt University Medical Center Institutional Animal Care and Use Committee and were performed in accordance with relevant guidelines and regulations. Mice harboring the heterozygous V408A mutation (V408A/+) were generously provided by Dr. James Maylie (Oregon Health & Science University)^34^. At approximately 6 weeks of age, male V408A/+ and wild-type littermates were euthanized by cervical dislocation under anesthesia followed by decapitation. Six brain regions (cerebellum, hippocampus, hypothalamus, cortex, striatum, olfactory bulb) and spinal cord were dissected from each mouse. Tissues were flash-frozen in liquid nitrogen and RNA was isolated by sonication in TRIzol (Ambion) according to the manufacturer’s instructions. RNA was reverse-transcribed and Kv1.1 editing was quantified by high-throughput sequence analysis as described for *in vitro* RNA editing analyses.

### Electrophysiological recording in Xenopus oocytes

All animal care and experimental procedures involving *Xenopus laevis* were approved by the University of Puerto Rico Institutional Animal Care and Use Committee and were performed in accordance with relevant guidelines and regulations. Kvβ1.1 and full-length, wild-type, V404I, I407M, and V408A Kv1.1 RNAs were transcribed *in vitro*, capped, and polyadenylated using the T7 mScript Standard mRNA Production System (CELLSCRIPT). Ovary sections containing several hundred oocytes were removed from adult specimens of *Xenopus laevis* obtained from Xenopus Express (Brooksville, FL). Oocytes were dispersed with type II collagenase and manually defolliculated. Stage V and VI oocytes were then selected by manual inspection for subsequent RNA injection. On day 1, oocytes were injected with 38.6 nL of one of the eight full-length Kv1.1 RNAs encoding edited and non-edited isoforms of wild-type, V404I, I407M, and V408A channels, with or without the Kvβ1.1 RNA. Injection concentrations were optimized individually for each construct, with greater concentrations required for the I407M and V408A RNAs due to protein expression differences previously described in the literature^21^,^22^. Each α-subunit was injected at a concentration from 2 ng/μL to 1 μg/μL and co-injected with Kvβ1.1 when applicable; concentrations for the Kvβ1.1 constructs were 10-fold more than each α-subunit, up to a maximum injection concentration of 500 ng/μL. Electrophysiological analysis of oocytes were performed between day 3–5 post-injection using the cut-open oocyte voltage-clamp technique^46^. The external solution consisted of: 20 mM K-glutamate, 100 mM L-glutamate, 2.5 mM MgCl_2_, 2.5 mM CaCl_2_, 10 mM HEPES, pH 7.4. The internal solution consisted of: 120 mM K-glutamate, 2.5 mM EGTA, 10 mM HEPES, pH 7.4. The pH of the solutions was adjusted using N-methyl-D-glucamine, as an alternative to NaOH, to limit the introduction of sodium ions into the solutions. To gain electrical access to the oocyte interior, the internal solution was supplemented with 0.3% saponin and used for a brief permeabilization prior to recording. The oocyte membrane potential was controlled using a CA-1B High Performance Oocyte Clamp (Dagan Corporation). Analog current signals were digitized at 100 kHz using an SBC6711 A/D D/A board (Innovative Integration, Simi Valley CA) and filtered at 5 kHz. To avoid errors introduced by series resistance, only traces exhibiting less than 10 μA were used for analysis. GPATCH M software, kindly provided by Dr. F. Bezanilla (University of Chicago), was used for data collection and clamp control. Leak currents were subtracted using a linear P/4 procedure. Data were analyzed using ANALYSIS software, also provided by Dr. F. Bezanilla, for fitting data with exponential functions and measuring current amplitudes. In addition, single exponential curves were fitted to recovery from the inactivation data using Graphpad Prism (Graphpad Software) to determine the rate constant, τ. As the channels encoded by edited V408A transcripts closed too rapidly for measurements of tail current amplitude, conductance (G) was calculated using Ohm’s law, equation (1),


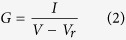


where I represents the maximal current at the test potential (V) and V_r_ signifies the reversal potential, determined empirically. For Figs 3, 4, 5, 6 and Supplementary Figures S5 and S7, points arising from brief capacity transients were removed for clarity.

### Statistical analysis

Statistical comparisons for *in vitro* (Fig. 1b) and *in vivo* (Fig. 2) editing analyses were determined by 2-way ANOVA with Tukey’s multiple comparisons test. Boltzmann functions were fitted using non-linear regression to model conductance-voltage curves (Fig. 3 and Supplementary Fig. S1) and to determine V_1/2_ and k values associated with each replicate (Table 1). Two-sample Student’s t-tests were used to compare voltage-dependent parameters (Table 1), long pulse characterization with and without Kvβ1.1 (Supplementary Figs S5 and S7), and recovery from inactivation τ values (Fig. 6b and Supplementary Table S1). The above analyses were conducted using Graphpad Prism (Graphpad Software). To maintain the type I error rate for each experiment at 5%, a Bonferroni correction was applied to each test based on the number of comparisons within each experiment and statistical significance for any pair of treatment comparisons was redefined according to these adjusted p-values. For Table 1 and Supplementary Table S1, 10 comparisons were made and the significance was adjusted to p ≤ 0.005. For Supplementary Figure S5b, 42 comparisons were made and the significance was adjusted to p ≤ 0.0012. For Supplementary Figures S5c and S7b, 30 comparisons were made and the significance was adjusted to p ≤ 0.0017. Analysis of activation and deactivation (Fig. 4 and Supplementary Figs S2 and S4), and inactivation kinetics (Fig. 5 and Supplementary Fig. S6) were performed with linear mixed models using the natural log of the acquired data to better meet model assumptions. Individual group comparisons for p-values were based on the Wald tests of model-based predicted (least square) means and appropriate standard errors. Because these data indicate that the measurements of activation, deactivation, and β-inactivation were dependent on voltage, comparisons were made only between values obtained at the same voltage. Data are presented with their original scale to allow for easier interpretation and comparison with the existing EA1 literature. All statistical tests were two-sided and statistical significance was defined as p ≤ 0.05, unless a specified Bonferroni correction was applied.

## Additional Information

**How to cite this article**: Ferrick-Kiddie, E. A. *et al*. Mutations underlying Episodic Ataxia type-1 antagonize Kv1.1 RNA editing. *Sci. Rep.* **7**, 41095; doi: 10.1038/srep41095 (2017).

**Publisher's note:** Springer Nature remains neutral with regard to jurisdictional claims in published maps and institutional affiliations.

## Supplementary Material

Supplementary Figures

## Figures and Tables

**Table 1:** Voltage-dependence of activation.

	V_1/2_ (mV)			k (mV)		
WT N	−31.6 ± 1.7			16.0 ± 0.9		
WT E	−34.5 ± 2.3			12.3 ± 1.4		
V404I N	−9.5 ± 1.4	****	]+++	14.7 ± 1.1		
V404I E	−20.3 ± 1.5	***		14.2 ± 0.9		
I407M N	−1.9 ± 0.8	****		10.7 ± 0.2	***	]++
I407M E	2.5 ± 1.1	****		9.4 ± 0.2		
V408A N	−29.4 ± 0.9			9.1 ± 1.0	***	
V408A E	−23.6 ± 3.4			8.1 ± 0.4		

Voltage-dependence of activation was determined by fitting data to a Boltzmann function, equation (2), to determine the midpoint of channel activation (V_1/2_) and relative voltage sensitivity (k). All data are represented as mean ± SEM, n = 4–8 oocytes for each channel type. Edited (E) and non-edited (N) isoforms of the mutant channels were compared to WT E and WT N channels, respectively: ***p ≤ 0.001; ****p < 0.0001. All types of N channels were compared to their respective E channels: ^++^p ≤ 0.005; ^+++^p ≤ 0.001. Due to multiple comparisons, significance was set at p ≤ 0.005.
