# Supplementary material for: Mutations underlying Episodic Ataxia type-1 antagonize Kv1.1 RNA editing
Source: Sci Rep. 2017 Feb 20;7:41095. doi: 10.1038/srep41095 (PMC5316942; doi:10.1038/srep41095)
Supplement: Supplementary Figures [file srep41095-s1.pdf]

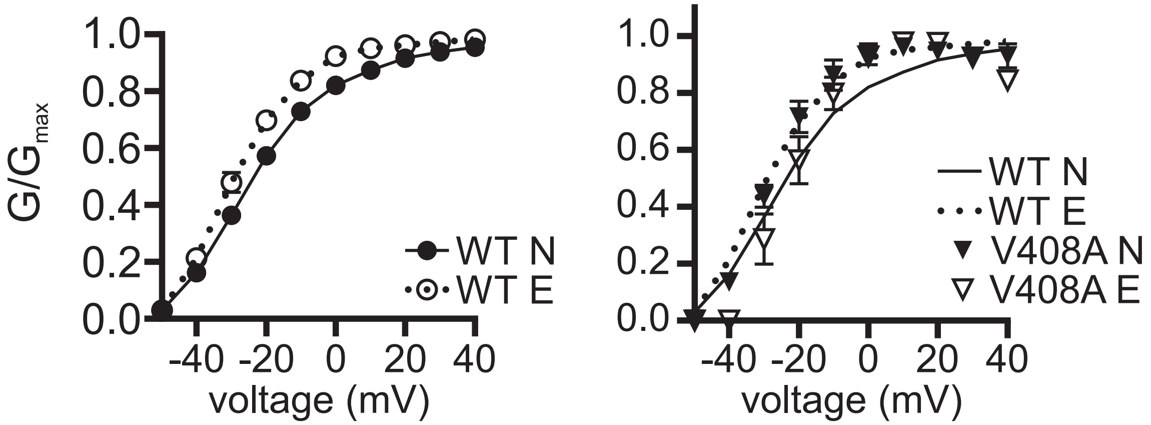

Supplementary Figure S1. Voltage-dependence of WT and V408A channels. Whole-cell K^+ currents were recorded from oocytes expressing either the non-edited (N) or edited (E) isoforms of the WT or V408A Kv1.1 channel. Test potentials were elicited in 10 mV voltage steps from -50 to 40 mV, from a holding potential of -80 mV. Normalized conductance was measured from tail current amplitude for all channel types except for V408A E, which exhibited tail currents too fast to measure and was derived by Ohm's Law, equation (1), as described in the *Materials and Methods*. Conductance (G) versus voltage plots were normalized to the maximal conductance (G_{max}) (mean ± SEM, n = 4-8 oocytes). Small error bars may be obscured by the data symbols in some cases.

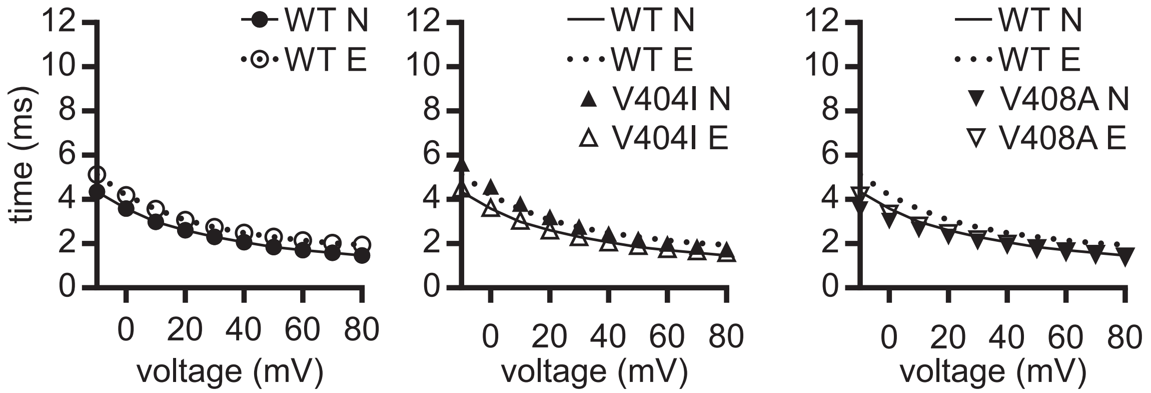

Supplementary Figure S2. Activation kinetics of WT, V404I, and V408A channels. Whole-cell K^+ currents were recorded from oocytes expressing either a non-edited (N) or edited (E) isoform of the wild-type (WT) or mutant Kv1.1 channel. Test potentials were elicited in 10 mV voltage steps from -10 to 80 mV, from a holding potential of -80 mV. Activation kinetics were measured as the time to reach half-maximal current amplitude (mean ± SEM, n = 3-7 oocytes). Small error bars were obscured by the data symbols.

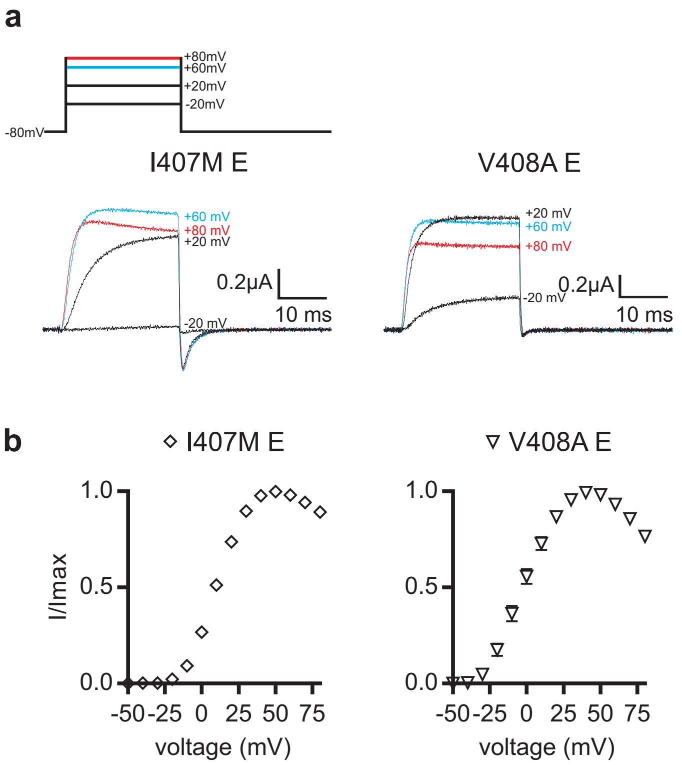

Supplementary Figure S3. I407M E and V408A E channels display decreased currents at the most positive voltages. Whole-cell K+ currents were recorded from oocytes expressing either the I407M E or V408A E Kv1.1 channel. Test potentials were elicited in 10 mV voltage steps from -50 to 80 mV, from a holding potential of -80 mV. (a) Representative traces for I407M E and V408A E channels are shown, indicating loss of current amplitude at the highest voltages. Voltages at 60mV (blue) and 80mV (red) are colored for clarity. (b) Current (I) versus voltage plots, normalized to the maximal current (Imax), are shown; note the unusual bell-shaped curves. Data points represent mean ± SEM (N=7-8 oocytes). Small error bars were obscured by the data symbols in some cases.

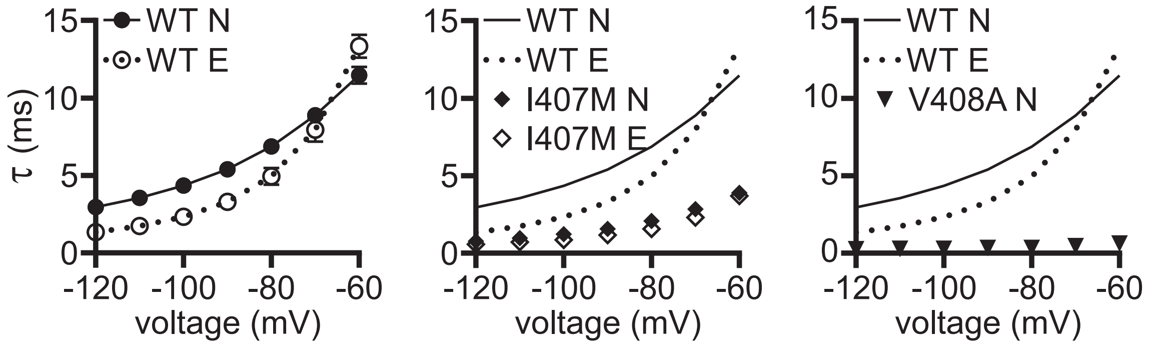

Supplementary Figure S4. Deactivation kinetics of WT, I407M and V408A channels. Whole-cell K^+ currents were recorded from oocytes expressing either a non-edited (N) or edited (E) isoform of the wild-type (WT) or mutant Kv1.1 channel. Following a holding potential of -80 mV and a depolarizing pulse to 20 mV, test potentials were elicited in 10 mV voltage steps from -120 to -60 mV. Closing kinetics were measured by fitting single exponential curves to the tail currents, to determine the associated τ value (mean ± SEM, n = 3-6 oocytes). WT N and WT E channels closed significantly slower than each other from -120 to -80 mV ($0.05 > p \geq 0.0002$). I407M E channels closed significantly faster than I407M N channels from -120 to -80 mV ($0.05 > p \geq 0.0036$). I407M N and I407M E channels closed significantly faster than their WT counterparts at all voltages tested (N: $p \leq 0.0001$; E: $0.01 > p \geq 0.0001$). V408A E channels closed too quickly for measurement, but V408A N channels closed significantly faster than WT N channels at all voltages ($p \leq 0.0001$). Small error bars were obscured by the data symbols in some cases.

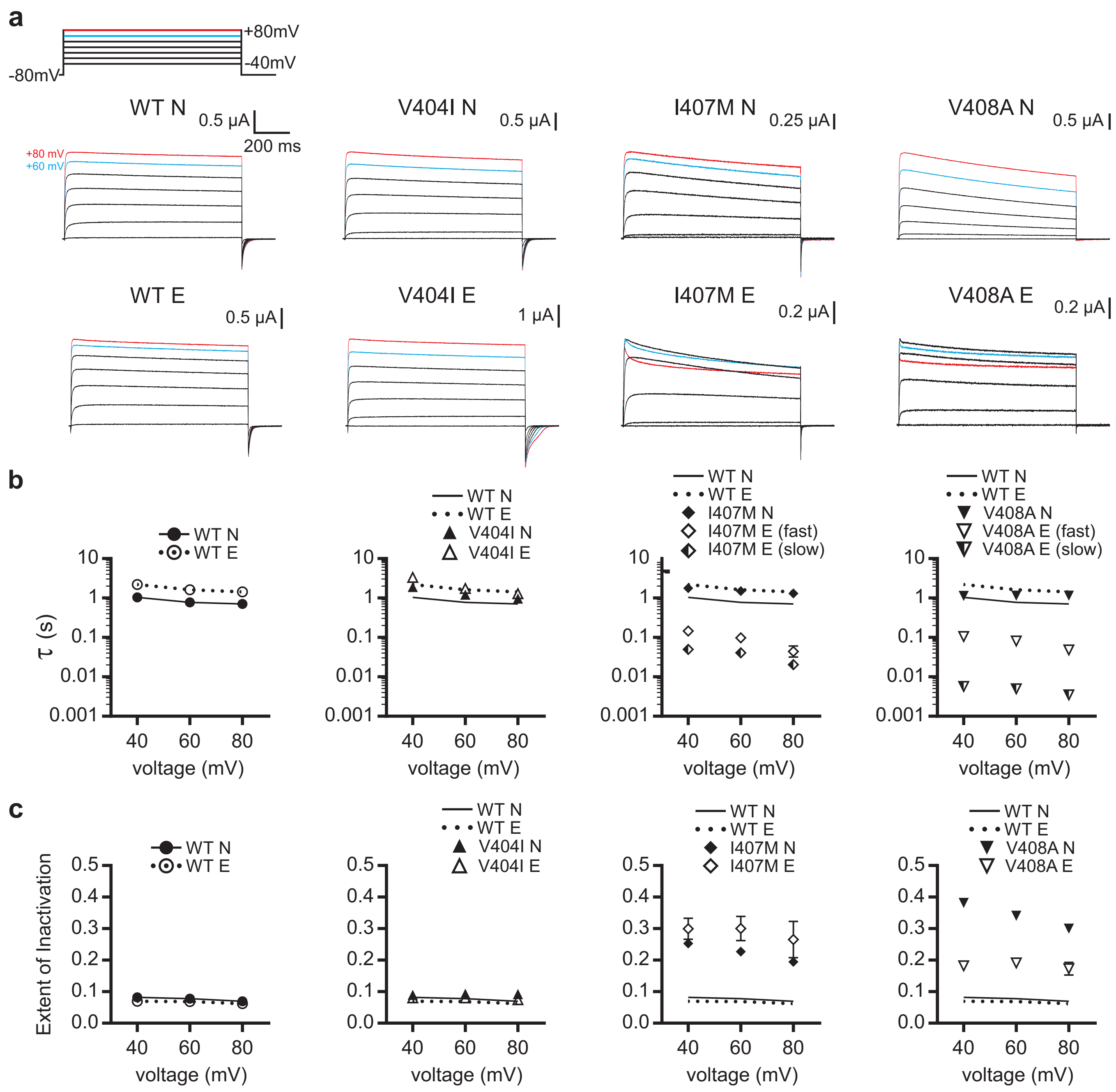

Supplementary Figure S5. Long pulse characterization of WT and EA1 channels. (a) Representative traces, depicting whole-cell K+ currents, were recorded from oocytes expressing either WT, V404I, I407M, or V408A channels, in the N or E isoform. Test potentials were elicited in 20 mV voltage steps from -40 to 80 mV for 1 s, from a holding potential of -80 mV. Voltages at 60mV (blue) and 80mV (red) are colored for clarity. (b) The slow inactivation was predominantly measured by fitting single exponential curves to the test pulse currents to determine τ values, however the I407M E and V408A E channels were best fit by double exponential curves with fast and slow τ values (mean ± SEM, n = 6-7 oocytes). Both the fast and slow τ values of I407M E were significantly faster than I407M N channels at all voltages tested. I407M N channels were significantly different from WT N from 60 to 80mV (though within the range of WT E). Both the fast and slow τ values of V408A E were significantly faster than V408A N channels at all voltages tested. (c) Extent of inactivation was measured by dividing the amplitude of inactivation by the total amplitude (mean ± SEM, n = 3-7 oocytes). I407M N channels had significantly greater inactivation that WT N channels at all voltages tested. V408A N channels had significantly increased inactivation compared to V408A E at all voltages tested. V408A N had significantly greater inactivation that WT N at all voltages tested and V408A E was significantly different from WT E channels from 40 to 60mV. Due to multiple comparisons, significance was set at $p \leq 0.0012$ for (b) and $p \leq 0.0017$ for (c). Small error bars were obscured by the data symbols in some cases.

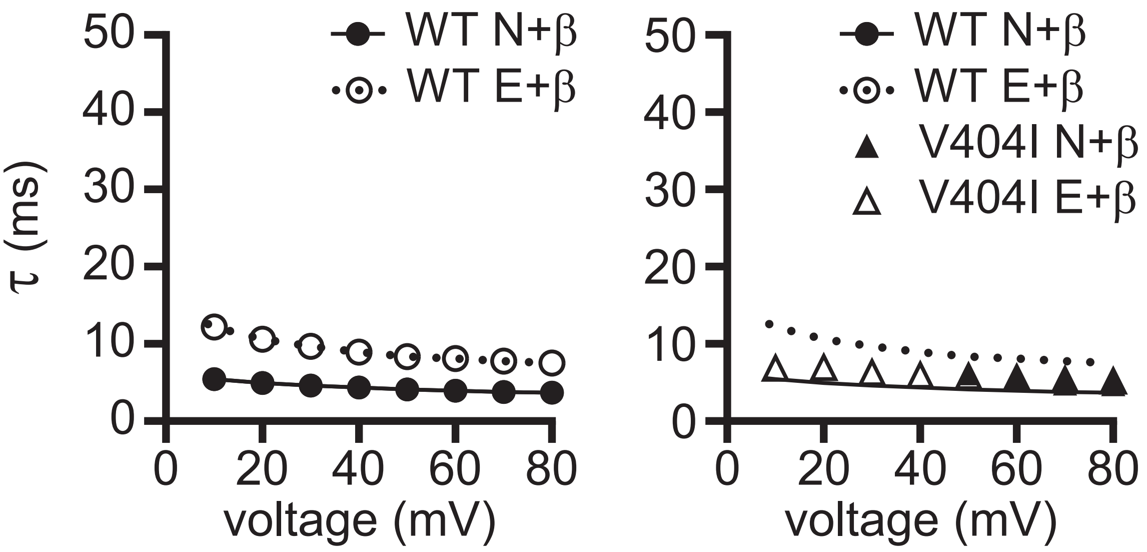

Supplementary Figure S6. Kvβ1.1-inactivation kinetics of WT and V404I channels. Whole-cell K+ currents were recorded from oocytes co-expressing the Kvβ1.1 subunit and either a WT or V404I channel, in the N or E isoform. Test potentials were elicited in 10 mV voltage steps from 10 to 80 mV, from a holding potential of -80 mV. Inactivation kinetics were measured by fitting single exponential curves to the test pulse currents, to determine the associated τ value (mean ± SEM, n = 3-6 oocytes). WT E channels were significantly slower than WT N channels at every voltage ($p \leq 0.0001$). V404I N and V404I E were not significantly different from each other, though each was significantly different from their respective WT counterpart (N: $0.05 > p \geq 0.0052$; E: $0.01 > p \geq 0.0002$). Small error bars were obscured by the data symbols.

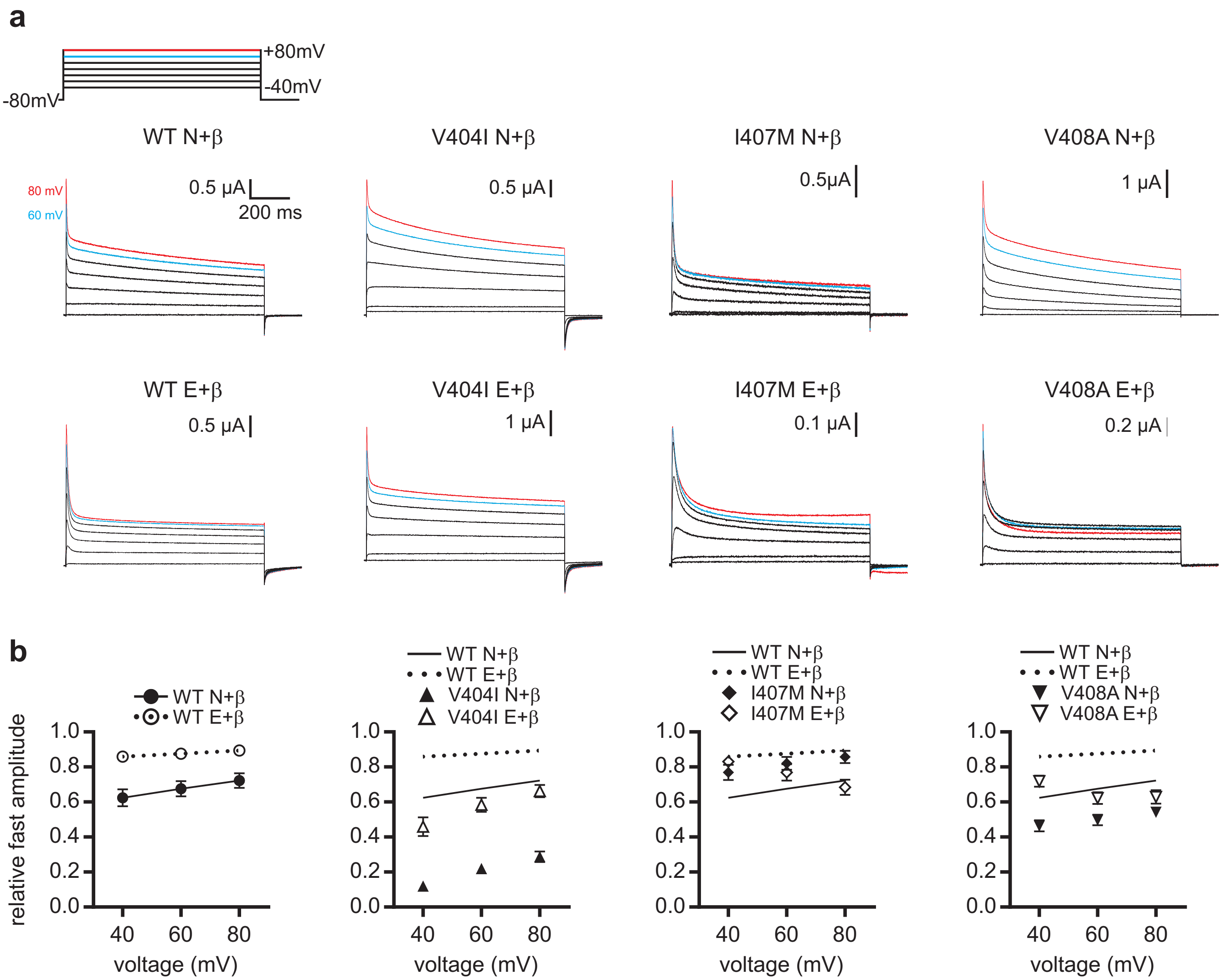

Supplementary Figure S7. Long pulse characterization of Kvβ1.1-inactivation of WT and EA1 channels. (a) Representative β-inactivation traces, depicting whole-cell K+ currents, were recorded from oocytes co-expressing the Kvβ1.1 subunit and either a WT, V404I, I407M, or V408A channel, in the N or E isoform. Test potentials were elicited in 20 mV voltage steps from -40 to 80 mV for 1 s, from a holding potential of -80 mV. Voltages at 60mV (blue) and 80mV (red) are colored for clarity. (b) The slow and fast inactivation was measured by fitting double exponential curves to the test pulse currents to determine the relative amplitude of the fast component of the inactivation (fast amplitude as a ratio of the total inactivation amplitude) (mean ± SEM, n = 5-7 oocytes). Due to multiple comparisons, significance was set at $p \leq 0.0017$. WT E channels were significantly slower than WT N channels from 40 to 60mV. V404I N and V404I E were significantly different from each other at every voltage tested, as well as compared to their respective WT counterpart. I407M N and I407M E channels were not significantly different from each other, though I407M E was significantly lower than WT E at 80mV. V408A N and V408AE channels only differed from one another at 40mV, and V408A E channels differed from WT E at 60 and 80mV. Small error bars were obscured by the data symbols in some cases.

	τ (ms)		fold change N vs. E
WT N+β	1912 ± 68.55	++++	16.8
WT E+β	113.6 ± 10.46		
V404I N+β	4218 ± 1189	+++	7.8
V404I E+β	538.0 ± 36.29 ****		
I407M N+β	783.2 ± 85.15 ****	++++	12.3
I407M E+β	63.82 ± 2.887 ***		
V408A N+β	388.4 ± 20.20 ****	++++	3.3
V408A E+β	80.83 ± 5.709		

Supplementary Table S1: Kinetics of recovery from Kvβ1.1 inactivation. All data represent mean ± SEM, n=3-7 oocytes for each channel type. Mutant N and E channels were compared to their WT N and E channels, respectively: ***p ≤ 0.001; ****p ≤ 0.0001. All types of N channels were compared to their respective E channels: +++p ≤ 0.001; ++++p ≤ 0.0001. Due to multiple comparisons, significance was set at p ≤ 0.005.
